# How the threat of climate change inspired my career in medicine (and I'm not the only one)

**DOI:** 10.1016/j.ebiom.2023.104689

**Published:** 2023-07-03

**Authors:** Harleen Marwah

**Affiliations:** The Children's Hospital of Philadelphia Division of General Pediatrics, United States

I was 15 years old the first time I remember the air quality was so dangerous that I could not play outside. I grew up in a region in Southern California with some of the lowest air quality ratings in the country. Though I did not understand it at the time, I experienced the influence of climate change on health and my community from a young age: from the annual threat of wildfires to persistent droughts. These lessons fueled my desire for the next generation to live in a healthier world and inspired my decision to go into pediatrics. In caring for children, I see the world through their eyes and cannot ignore how the changing environment shapes their health.

My own perspectives have been shaped by my experiences as a first-generation American growing up in a Sikh family. My mother grounded me in the Indian concept of “seva,” a selfless service performed without expectation of anything in return. My grounding in seva and my passion for the sciences led me to study the social determinants of health. After my graduate program in Global Medicine, I was inspired to advocate for the health consequences of climate change on an international stage. At the United Nations Framework Convention on Climate Change 21st Conference of Parties, I facilitated a series of conversations focused on intergenerational equity. I experienced how health cuts across boundaries and is a powerful tool to open meaningful dialogue. By bringing together delegates who might not otherwise have interacted, important partnerships were formed including one between an agriculture team from South America and a health research team from England. Furthermore, at these meetings, I observed how physicians advocated for their patients and elevated voices that were not traditionally represented at decision making tables, especially those of children.

Coming to medical school, I understood that climate change was a health emergency and I saw firsthand how climate change was affecting my patients. I met a mother of five young children, all of whom suffered from moderate to severe asthma and frequented the hospital's emergency department. She shared how she had taught all her children to read the air quality index so they could determine if it was safe to play outside. Witnessing firsthand how the health of the patients I cared for is influenced by the changing environment, I was inspired to do more.

I was motivated by the framework of problem-solving we learned in training: collaboration. Our toughest clinical cases require a team of experts, from diverse backgrounds and disciplines. We trusted this model to save lives and I was energized to scale this framework to tackle climate change in healthcare. I founded Medical Students for a Sustainable Future (MS4SF) in 2019 to unite medical students who recognize the urgency of the climate crisis to health and social justice and are inspired to catalyze change now. In this community, students engage in work through advocacy, research, climate-smart healthcare, medical education, and more. MS4SF allows change-making to be accessible to students and there is space for students to learn, engage, and organize. MS4SF members build a solid foundation in this work and develop valuable skills for their careers including coalition-building, public speaking, and narrative medicine, among others. In its first year, the MS4SF community grew to over 100 students nationwide through grassroots efforts. Today, the community welcomes over 700 students from over 130 schools. In each year, I meet even more students who were inspired to become doctors due to the health impacts of climate change. The eagerness and enthusiasm of medical students to join the movement reflects a deep understanding that climate change is a critical and urgent issue for healthcare.

It has been over ten years since that first time the air quality prevented me from playing outside, but these negative effects have not been reversed and people are demanding change. The next generation is fighting for their future: from organizing strikes and school walk-outs to taking legal action against governments. My oath in medicine to “do no harm” inherently applies to the clinical setting. When it comes to the health crisis of climate change, it is apparent this oath also applies to the role of health professionals as communicators, advocates, and leaders. In the first MS4SF video message, we explain, “On the other side of crisis, there is opportunity; there is resilience; there is hope.” Tackling the climate crisis requires community and collaboration, which generates my favorite renewable resource: hope.

## Declaration of interests

HM does not have any conflict of interest to report.

